# Combined maximum *b*‐value and echo time: A practical method for determining the signal‐to‐noise ratio for magnetic resonance images

**DOI:** 10.1002/acm2.13497

**Published:** 2021-12-21

**Authors:** Naoki Ohno, Tosiaki Miyati, Hirotaka Oyabu, Toshifumi Gabata, Satoshi Kobayashi

**Affiliations:** ^1^ Faculty of Health Sciences, Institute of Medical, Pharmaceutical and Health Sciences Kanazawa University Kanazawa Japan; ^2^ Department of Radiology Kanazawa University Hospital Kanazawa Japan

**Keywords:** diffusion, phantom, signal‐to‐noise ratio, T_2_ relaxation time

## Abstract

**Purpose:**

The aim of the present study is to develop a simple and practical method for measuring the signal‐to‐noise ratio (SNR) of magnetic resonance images called combined maximum *b*‐value and echo time (COMBET) that could be suitable for pulse sequences to which a diffusion gradient can be applied.

**Methods:**

In the COMBET method, we first obtain a signal image using the objective pulse sequence. Then, we obtain the noise image of this sequence using the diffusion gradient with the largest *b*‐value and longest echo time. However, other imaging parameters are the same as those used for the signal image acquisition. The SNR is calculated from the mean signal intensity in the region of interest (ROI) of the signal image divided by the signal standard deviation in the ROI of the noise image after the required corrections. We compared SNRs determined using the COMBET and double echo with the longest second echo time (DELSET) methods for single‐shot echo‐planar imaging and fast spin‐echo sequences in white mineral oil phantom, purified water phantom, human head, and upper abdomen. We used the subtraction method as the reference standard.

**Results:**

The COMBET method could obtain the optimal noise image, whereas the DELSET method could not sufficiently suppress the long T_2_ signal in the purified water phantom, cerebrospinal fluid, and digestive fluid. Therefore, the DELSET method afforded incorrect results for the long T_2_ regions in the noise and SNR maps, while the COMBET method enabled the in vivo evaluation of the SNR even in the long T_2_ regions.

**Conclusion:**

The COMBET method allows simple and practical SNR measurement, which is applicable to tissues with long T_2_ relaxation time.

## 1 INTRODUCTION

The signal‐to‐noise ratio (SNR) of magnetic resonance imaging (MRI) is a vital parameter for the quality assurance and control of the obtained magnetic resonance (MR) images, optimization of imaging parameters, or evaluation of image quality in clinical MR images.^1^, ^2^ Although several methods, including the background and image subtraction methods, have been used to measure the SNR of MR images, these methods are limited by the measurement conditions.^3^, ^4^ In the background method, the noise is estimated by applying an appropriate correction to the standard deviation (SD) of signals in the region of interest (ROI) in the background area outside the image object for a magnitude operation.^3^, ^4^ This is the simplest measurement method and thus has been widely used in the SNR evaluation of clinical MR images. However, this method is not valid when parallel imaging or image nonuniformity correction is used because that causes the noise estimation to vary spatially depending on the measurement position.^5^ In the image subtraction method, two images obtained under identical imaging conditions are subtracted to create a noise image. Then, the noise is estimated by applying an appropriate correction to the SD of signals in the ROI in the noise image for the subtraction process.^6^ This method is considered the gold standard for the SNR measurement of phantoms and is applicable even when parallel imaging or image nonuniformity correction is used. However, because it requires two consecutive scans with identical imaging conditions, an erroneous SNR can be caused by the high sensitivity to system drift and image misregistration between the scans.^5^, ^7^ Therefore, this method is not necessarily valid for in vivo MR images in clinical practice.

In a recent study, a novel method for simply and accurately measuring the SNR was developed called double echo with the longest second echo time (DELSET).^8^ This method is based on the double‐echo sequence. The first echo time (TE) is set at the clinically used time for the signal image, and the second TE is set at the longest time in the sequence for the noise image. The second TE needs to be at least eight times longer than T_2_ of tissues or materials such that the signals are sufficiently attenuated to the noise level due to the T_2_ relaxation process. However, the DELSET method can generate errors in the noise estimation owing to residual signals in the noise image. These residual signals originate from long T_2_ tissues (e.g., including the cerebrospinal fluid [CSF] and digestive fluid) due to the limit on the maximum available TE in MRI systems.

The aim of this study was to develop a novel method that combines the DELSET method with a diffusion gradient to address the effect of long T_2_ tissue on SNR measurement, which we termed combined maximum *b*‐value and echo time (COMBET).

## 2 METHODS

### 2.1 COMBET method

**FIGURE 1:**
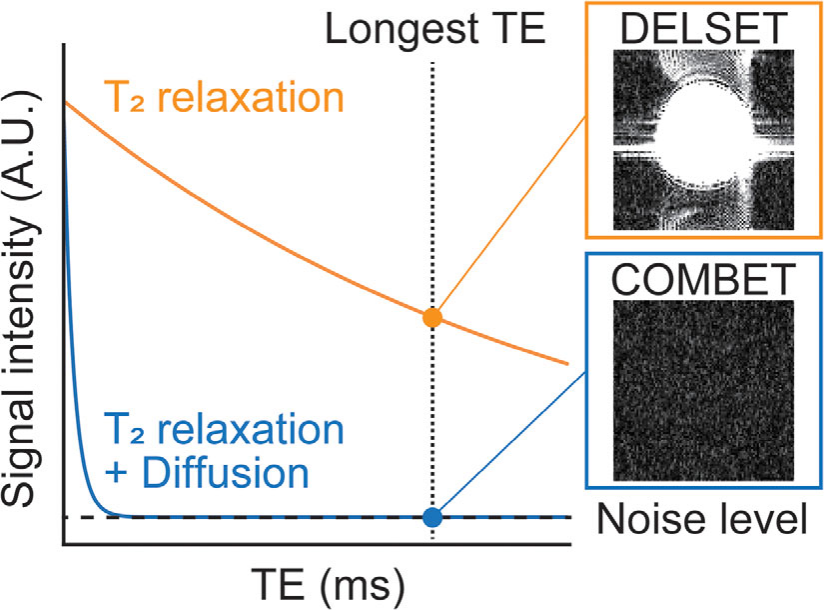
Overview of noise image acquisition in a purified water phantom with long T_2_ values when using the COMBET andDELSET methods. With the COMBET method, the signal intensity in the acquired noise image reaches the noise level even in the long T_2_ phantom because the signal attenuation is accelerated by using both the diffusion gradient with the largest *b*‐value and the longest TE. By contrast, the signals remain with the DELSET method because of insufficient signal attenuation. Abbreviations: COMBET, combined maximum *b*‐value and echo time; DELSET, double echo with the longest second echo time; TE, echo time

The COMBET method is based on pulse sequences in which the diffusion gradient can be applied. We obtained a signal image using the objective pulse sequence and noise image utilizing the diffusion gradient with the largest *b*‐value and longest TE (Figure 1). Imaging parameters except for the *b*‐values and TE were kept constant between the signal and noise image acquisitions. Then, we determined the mean signal intensity in an ROI in the signal image and the SD of signals for the same ROI in the noise image. Considering the effects of magnitude operation and multichannel coil image reconstruction,^3^, ^4^, ^9^ the following correction was applied to the SD^10^:

(1) $$\;SD_{c}=\;\mathrm{SD}/\sqrt{2N-\beta {\left(N\right)}^{2},}$$

where SD_c_ is the corrected SD, SD is the measured SD in an ROI in a noise image, and *N* is the number of coil channels. β(N) is defined as follows:

(2) $$\beta \left(N\right)\;=\;\sqrt{\pi \;/\;2}\cdot \left[\left(2N-1\right)!!\right]/\left[2^{N-1}\cdot \left(N-1\right)!\right],$$

where the symbols ! and !! denote factorial and double factorial, respectively. Finally, the SNR was calculated from the mean signal intensity divided by the corrected SD.

### 2.2 DELSET method

By the DELSET method, we obtained the noise image using the longest TE but without the diffusion gradient. The mean signal intensity and SD of signals were determined in the ROIs in the signal and noise images, respectively, in the same manner as for the COMBET method. An appropriate correction was applied to the SD as with the COMBET method. Then, the SNR was calculated by dividing the mean signal intensity by the corrected SD.

### 2.3 Subtraction method

We used the subtraction method as the reference standard for determining the SNR. The difference image was obtained by subtraction of two signal images obtained under identical imaging conditions. The SD of the signals in the ROI in the difference image (SD_sub_) was determined in the same manner as in the COMBET and DELSET methods. Then, the noise was estimated by dividing the noise SD by an appropriate correction factor, given as follows^6^:

(3) $$\mathrm{Noise}\;=\;SD_{\mathrm{sub}}\;/\sqrt{2},$$

where $\sqrt{2}$ is the correction factor for the bias caused by the image subtraction process. The SNR was calculated by dividing the mean signal intensity in the ROIs in the two signal images by the estimated noise.

### 2.4 Phantom experiments

**FIGURE 2:**
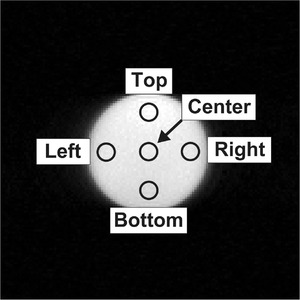
Example of five region of interest sets on a phantom image

We conducted phantom experiments using a 3.0‐T MRI system with a 16‐channel phased‐array head‐neck coil (Ingenia, Philips Healthcare, Best, The Netherlands). We prepared two types of uniform cylindrical phantoms: One filled with white mineral oil (T_1_, T_2_, and the diffusion coefficient of 196, 75, and 0.04 × 10^−3^ mm^2^/s, respectively) and the other filled with purified water (T_1_, T_2_, and the diffusion coefficient of 3505 ms, 2390 ms, and 2.1 × 10^−3^ mm^2^/s, respectively). The T_1_ and T_2_ of the phantoms were determined using a multi‐echo spin‐echo interleaved with a multi‐echo inversion recovery sequence.^11^ The diffusion coefficients of the phantom were determined using single‐shot echo‐planar imaging (SSEPI) with *b*‐values of 0 and 1000 s/mm^2^. The imaging parameters are summarized in Table 1. The diameter and length of the phantoms were 10 and 9 cm, respectively. These phantoms were scanned using two different pulse sequences: SSEPI and fast spin‐echo (FSE). A diffusion gradient was applied to both sequences. The single‐slice SSEPI and FSE sequences were applied using the imaging parameters shown in Table 1. The SSEPI scans were repeated 20 times to allow for statistical comparison and to evaluate the variability of the measured SNR. For both the sequences, the diffusion gradient was separately applied in three orthogonal directions (x, y, and z) and two consecutive scans were conducted under identical imaging conditions for the subtraction method. Then, we placed five circular ROIs (center, top, bottom, right, and left) in the signal and noise images of the phantom as shown in Figure 2. Each ROI had a size of 52 pixels. Statistical analyses were performed with SPSS Statistics (version 25, IBM, Armonk, NY, USA). We compared the SNRs measured using COMBET and DELSET with Student's *t*‐test with Bonferroni correction for five multiple comparisons. We used the subtraction method as the reference standard. Significance was set at a *p*‐value < 0.05. Moreover, coefficient of variation (CV) of the SNR in the white mineral oil phantom was calculated by dividing the SD of the measured SNR by the mean of 20 consecutive SSEPI scans to evaluate variability and to perform comparisons among the COMBET, DELSET, and subtraction methods.

**TABLE 1:** Imaging parameters for phantom and in vivo experiments

	T_1_ and T_2_ for phantom	Diffusion for phantom	SNR for phantom and head		SNR for abdomen
Receive coil	16‐channel phased‐array head‐neck coil		16‐channel phased‐array head‐neck coil		32‐channel phased‐array body coil
Pulse sequence	Multi‐echo spin‐echo interleaved with multi‐echo inversion recovery sequence	SSEPI	SSEPI	FSE	SSEPI
TE (ms)	30, 60, 90, and 120	72	37 for signal; 500 for DELSET and COMBET	29 for signal; 275 for DELSET and COMBET	46 for signal; 500 for DELSET and COMBET
TR (ms)	2300 for spin‐echo; 920 for inversion recovery	4000	3000	8000	3000
TI (ms)	500	–	–	–	–
FOV (mm)	256	256	256	256	360
Imaging matrix	128 × 128	128 × 128	128 × 128	256 × 256	128 × 128
Flip angle (°)	90	90	90	90	90
Slice thickness (mm)	5	5	5	5	5
NSA	2	1	1	1	1
rBW (Hz/pixel)	85	1882	1864	957	2032
*b*‐Value (s/mm^2^)	–	0 and 1000	0 for signal and DELSET; 18000 for COMBET	0 for signal and DELSET; 18000 for COMBET	0 for signal and DELSET; 18000 for COMBET
SENSE factor	–	2.0	–	–	2.0
Scan time	13 min 48 s	44 s	6 s each for signal, DELSET, and COMBET	16 s each for signal, DELSET, and COMBET	6 s each for signal, DELSET, and COMBET

**Abbreviations**: COMBET, combined maximum *b*‐value and echo time; DELSET, double echo with the longest second echo time; FOV, field of view; FSE, fast spin‐echo; NSA, number of signals averaged; rBW, receive bandwidth; SENSE, sensitivity encoding; SNR, signal‐to‐noise ratio; SSEPI, single‐shot echo‐planar imaging; TE, echo time; TI, inversion time; TR, repetition time.

For the obtained phantom images, signal and noise maps were created by calculating the local mean signal intensity of the signal image and corrected SD of the noise image in a 7 × 7 sliding window. The SNR map was calculated by dividing the signal map by the noise map on a pixel‐by‐pixel basis.

The white mineral oil phantom was scanned using the SSEPI and FSE sequences under the above‐mentioned imaging conditions, except that the slice thicknesses was varied from 1 to 6 mm in steps of 1 mm. We placed a circular ROI containing 1264 pixels at the center of the phantom image and calculated the SNRs using the COMBET, DELSET, and subtraction methods. Furthermore, to evaluate the linearity of SNR with increasing effective slice thickness, we scanned an International Electrotechnical Commission (IEC) standard phantom, which is the international standard for the measurement of effective slice thickness.^12^ Notably, the imaging conditions were kept the same as those used for the signal image acquisition in each sequence, except for a sensitivity encoding (SENSE) factor of two for reducing image geometric distortion. The effective slice thickness was calculated using a slab method according to the IEC standard.^12^ Next, we evaluated the linearity between the measured SNR using each method and the effective slice thickness.

### 2.5 In vivo experiments

The head and upper abdomen of a healthy volunteer were scanned to demonstrate the validity of the COMBET method in vivo. This study was approved by the appropriate institutional review board. The purpose and procedures of the study were fully explained to the subject, who provided written informed consent before the scans. Head scans of the subject were performed using the same MRI system and sequences (SSEPI and FSE) with the identical parameters used for the phantom experiments (Table 1). For the upper abdominal scans, we used a 32‐channel phased‐array body coil. The upper abdominal images of the patient holding breath were obtained using SSEPI with the imaging parameters shown in Table 1. For the obtained head and upper abdominal images, the signal, noise, and SNR maps were created in the same manner as in the phantom experiments.

## 3 RESULTS

**FIGURE 3:**
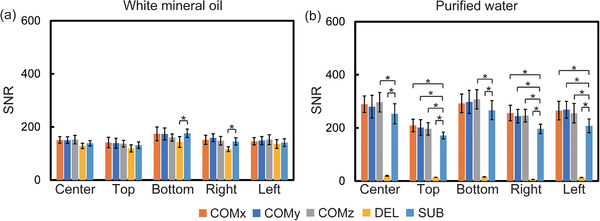
Comparison between SNRs obtained using the COMBET, DELSET, and subtraction methods with the SSEPI sequence in the (a) white mineral oil and (b) purified water phantoms. Error bars indicate the standard deviation of SNR over 20 measurements. Abbreviations: SNR, signal‐to‐noise ratio; SSEPI, single‐shot echo‐planar imaging; COM, COMBET; DEL, DELSET; SUB, subtraction. Note that x, y, and z denote the three orthogonal diffusion gradient directions used in the COMBET method. *p < 0.05

**FIGURE 4:**
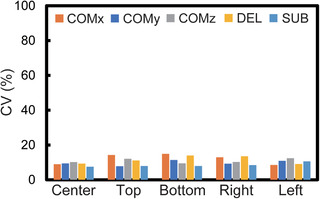
Comparison between the CVs of the signal‐to‐noise ratios obtained using the COMBET, DELSET, and subtraction methods with the single‐shot echo‐planar imaging sequence in the white mineral oil phantom. Abbreviations: CV, coefficient of variation; COMBET, combined maximum *b*‐value and echo time; DELSET, double echo with the longest second echo time; COM, COMBET; DEL, DELSET; SUB, subtraction. Note that x, y, and z denote the three orthogonal diffusion gradient directions used in the COMBET method

**FIGURE 5:**
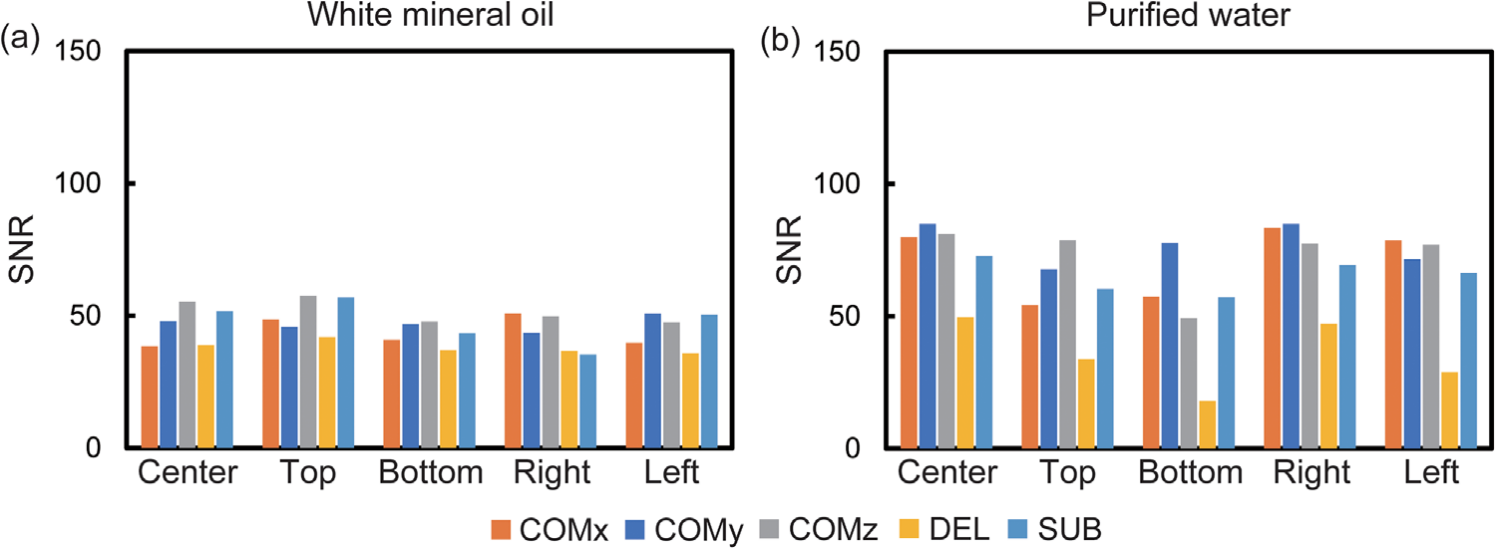
Comparison between the SNRs obtained using the COMBET, DELSET, and subtraction methods with the fast spin‐echo in the (a) white mineral oil and (b) purified water phantoms. Abbreviations: SNR, signal‐to‐noise ratio; COMBET, combined maximum *b*‐value and echo time; DELSET, double echo with the longest second echo time; COM, COMBET; DEL, DELSET; SUB, subtraction. Note that x, y, and z denote the three orthogonal diffusion gradient directions used in the COMBET method

**FIGURE 6:**
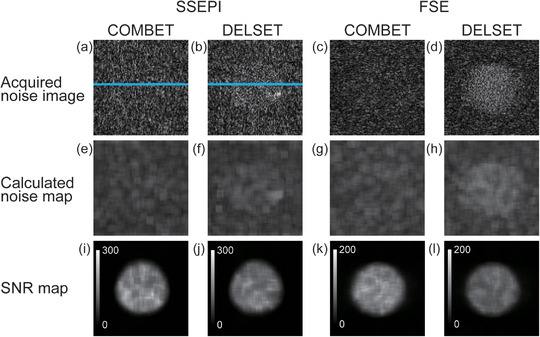
(a–d) Acquired noise images, (e–h) calculated noise maps, and (i–l) signal‐to‐noise ratio (SNR) maps of the white mineral oil phantom obtained using the (a, e, i, c, g, k) combined maximum *b*‐value and echo time (COMBET) and (b, f, j, d, h, l) double echo with the longest second echo time (DELSET) methods with the (a, b, e, f, i, j) single‐shot echo‐planar imaging (SSEPI) and (c, d, g, h, k, l) fast spin‐echo (FSE) sequences. Blue lines in (a–d) represent signal profiles

**FIGURE 7:**
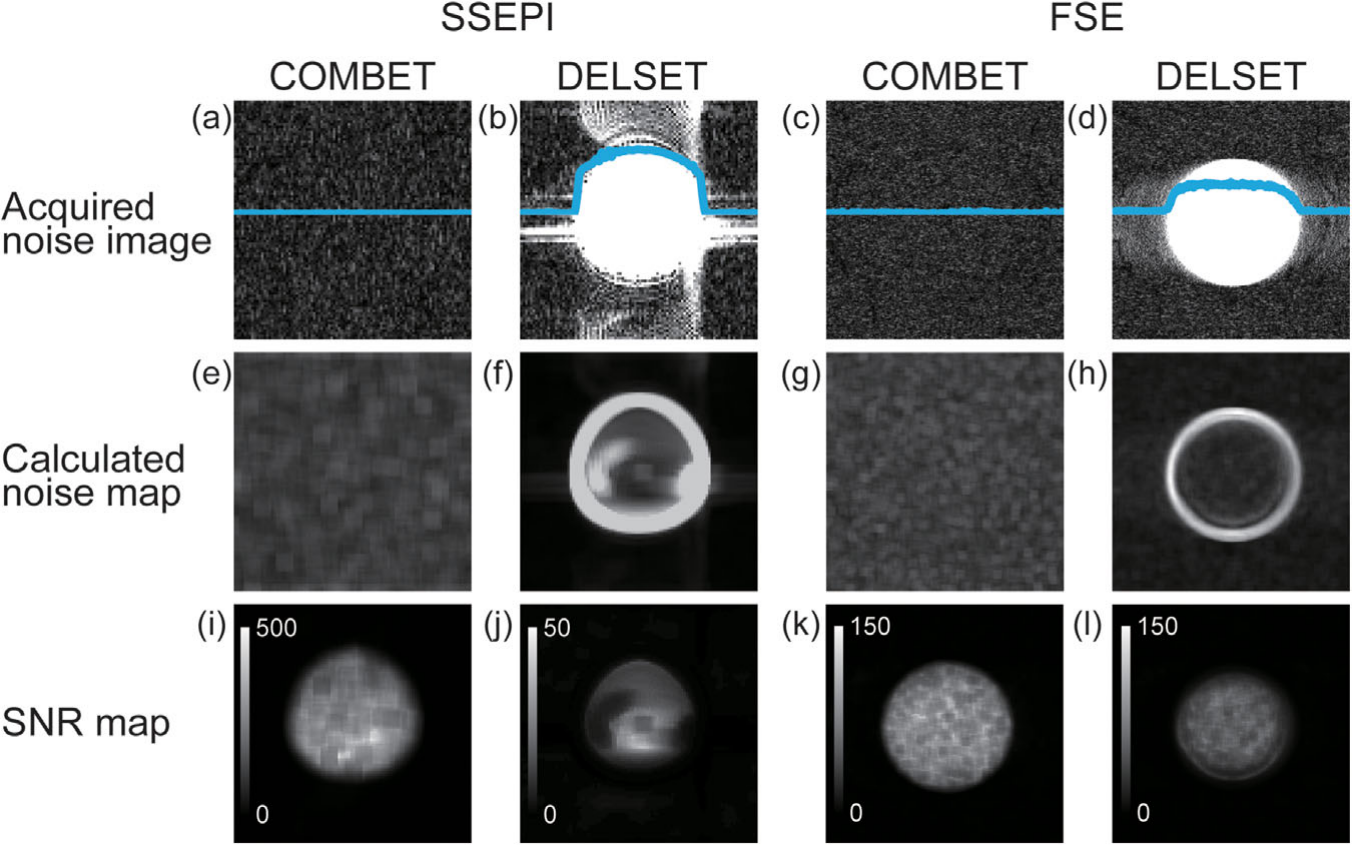
(a–d) Acquired noise images, (e–h) calculated noise maps, and (i–l) signal‐to‐noise ratio (SNR) maps of the purified water phantom obtained using the (a, e, i, c, g, k) combined maximum *b*‐value and echo time (COMBET) and (b, f, j, d, h, l) double echo with the longest second echo time (DELSET) methods with the (a, b, e, f, i, j) single‐shot echo‐planar imaging (SSEPI) and (c, d, g, h, k, l) fast spin‐echo (FSE) sequences. Blue lines in (a–d) represent signal profiles

Figure 3 shows the comparison of SNR measurements in the SSEPI sequence between the COMBET, DELSET and subtraction methods for the white mineral oil and purified water phantoms. Note that the SNR results obtained using the COMBET method were shown for each diffusion gradient direction. In the white mineral oil phantom, the SNRs obtained using the COMBET method were generally comparable to those obtained using the subtraction method as the reference standard, whereas the SNRs obtained using the DELSET method were lower, with statistically significant differences observed in the bottom and right ROIs (*p*‐values after correction for multiple comparison = 4.8 × 10^−5^ and 4.7 × 10^−7^ for the bottom and right ROIs, respectively). In the long T_2_ purified water phantom, the SNRs obtained using the COMBET method were higher than those obtained using the subtraction method, whereas those obtained using the DELSET method were substantially lower, with statistical significances observed in all ROIs (the maximum *p‐*value = 7.2 × 10^−36^ for the left ROI). Figure 4 shows the comparison of the CVs of the measured SNRs using each method in the SSEPI sequence. The CVs of the three methods were generally comparable. Figure 5 shows the comparison of SNRs in the FSE sequence between the methods for the same phantoms. The results of the white mineral oil phantom were similar to those of the SSEPI sequence. The SNRs obtained using the COMBET method were generally consistent with those obtained using the subtraction method even in the purified water phantom. Overall, the SNRs of the FSE sequence were lower than those of the SSEPI sequence in both phantoms. Figures 6 and 7 show the acquired noise image and calculated noise and SNR maps of the white mineral oil and purified water phantoms, respectively, which were obtained using the COMBET and DELSET methods in the SSEPI and FSE sequences. The noise image and noise map obtained using the COMBET method showed a uniform noise distribution, indicating that residual signals were not observed in the phantoms. In contrast, the images obtained using the DELSET method clearly showed enhanced noise due to the residual signals from the long T_2_ phantom especially in the purified water phantom, which resulted in an erroneous decrease in the SNR.

**FIGURE 8:**
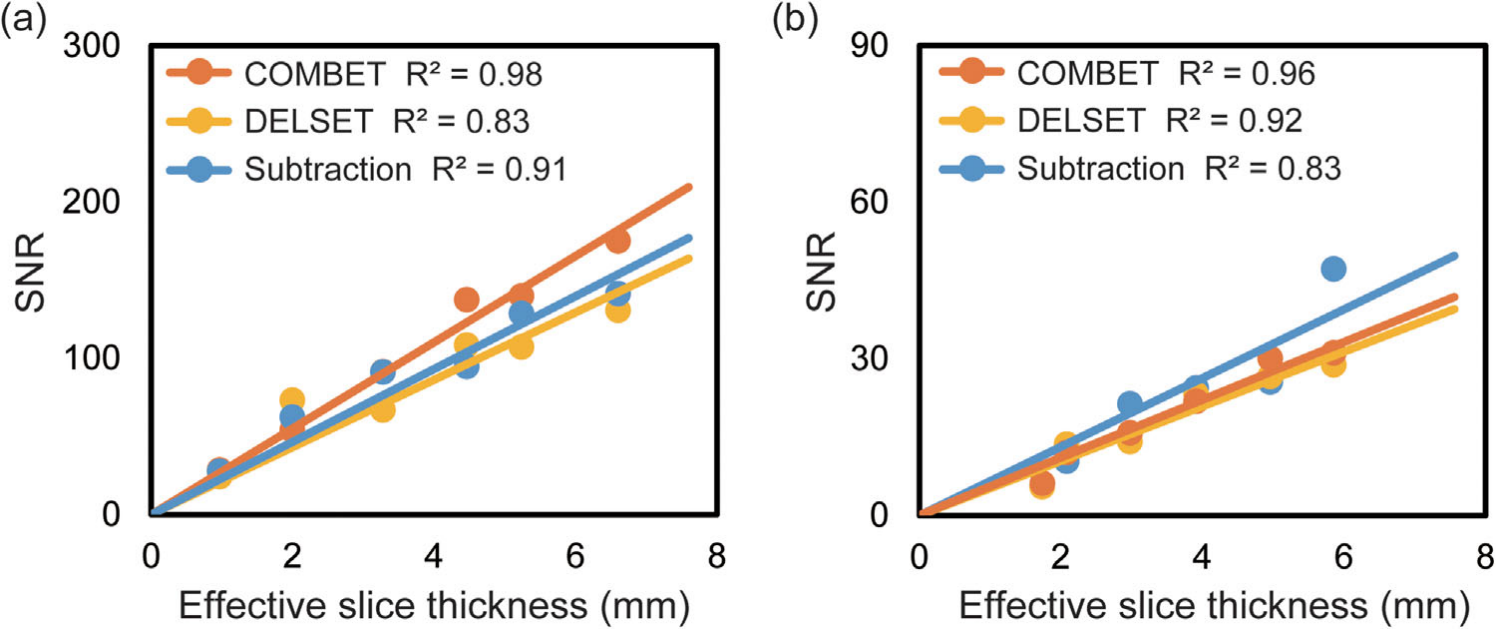
Scatterplots of the effective slice thickness versus signal‐to‐noise ratio (SNR) obtained using the combined maximum *b*‐value and echo time (COMBET), double echo with the longest second echo time (DELSET), and subtraction methods with the (a) single‐shot echo‐planar imaging and (b) fast spin‐echo sequences. *R* ^2^ represents the coefficient of determination

Figure 8 shows the scatterplots comparing the effective slice thickness and measured SNR obtained using each method in the SSEPI and FSE sequences. For both the sequences, excellent positive correlations between the effective slice thickness and SNR were observed when the COMBET method was used.

**FIGURE 9:**
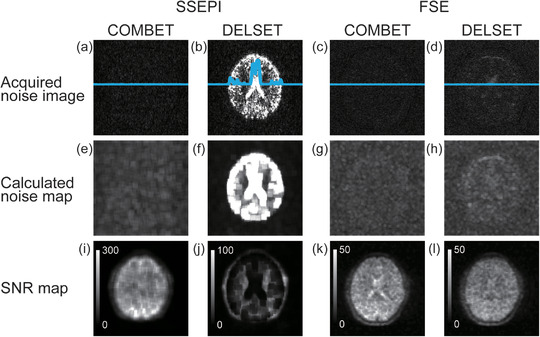
(a–d) Acquired noise images, (e–h) calculated noise maps, and (i–l) signal‐to‐noise ratio (SNR) maps of the head obtained using the (a, e, i, c, g, k) combined maximum *b*‐value and echo time (COMBET) and (b, f, j, d, h, l) double echo with the longest second echo time (DELSET) methods with the (a, b, e, f, i, j) single‐shot echo‐planar imaging (SSEPI) and (c, d, g, h, k, l) fast spin‐echo (FSE) sequences. Blue lines in (a–d) represent signal profiles

**FIGURE 10:**
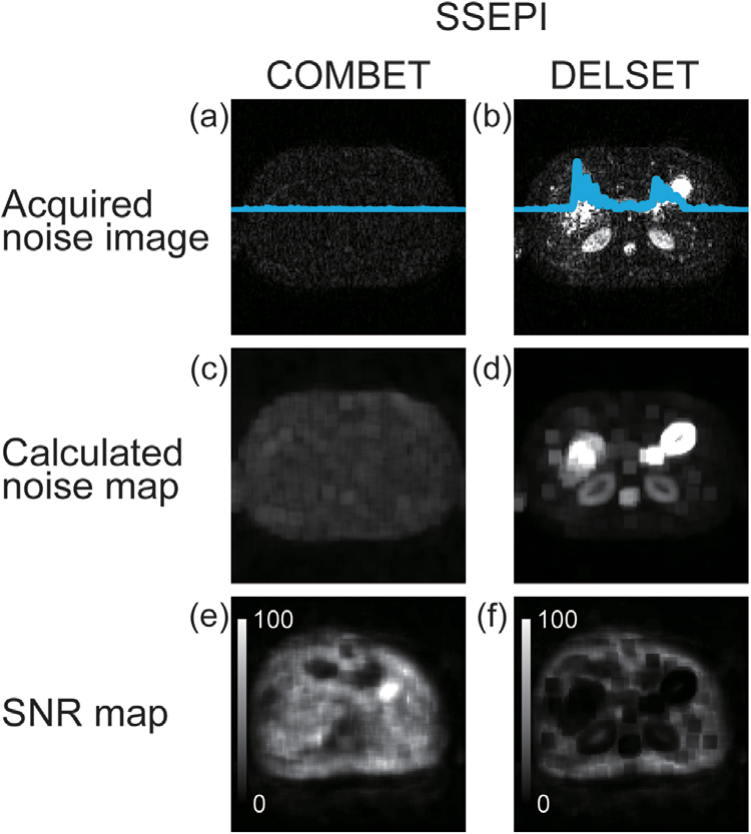
(a, b) Acquired noise images, (c, d) calculated noise maps, and (e, f) signal‐to‐noise ratio (SNR) maps of the upper abdomen obtained using the (a, c, e) combined maximum *b*‐value and echo time (COMBET) and (b, d, f) double echo with the longest second echo time (DELSET) methods with the single‐shot echo‐planar imaging (SSEPI) sequence. Blue lines in (a) and (b) represent signal profiles

Figures 9 and 10 show the acquired head and upper abdominal noise images and the calculated noise and SNR maps obtained using the COMBET and DELSET methods. Residual signals from tissues were not observed in the noise images and maps of the head and upper abdomen obtained using the COMBET method. By contrast, the DELSET method showed enhanced noise in the long T_2_ tissues, including the CSF and digestive fluid in the stomach, where the signal was present. Consequently, the SNR erroneously decreased in such tissues.

## 4 DISCUSSION

Using the DELSET method is a simple and accurate way of measuring the SNR for MRI.^8^ However, it is not applicable to tissues and materials with long T_2_ values. Therefore, the aim of this study was to develop the COMBET method to address this issue and evaluate this method's feasibility in the phantom and in vivo experiments.

In the experiments involving the white mineral oil phantom, the SNRs obtained using the COMBET method were comparable with those obtained using the subtraction method, which is considered the reference standard for SNR measurements.^6^ This validates the COMBET method. By contrast, the DELSET method showed slightly lower SNRs in both the sequences compared with the subtraction method. The difference can be attributed to the residual signals in the noise image when the DELSET method was used. This is because the TE (500 and 275 ms for the SSEPI and FSE sequences, respectively) was not set to be eight times longer than the T_2_ value of the white mineral oil phantom (75 ms) owing to the limited maximum TE available in the MRI system.^8^ Furthermore, the COMBET and subtraction methods resulted in comparable SNRs for the purified water phantom with a long T_2_ value (2390 ms) in the FSE sequence. This indicates that the signals in the noise image were sufficiently attenuated so that noise was the dominant effect even in the long T_2_ phantom owing to the diffusion and T_2_ relaxation processes. However, the COMBET and subtraction methods exhibited slightly different SNRs in the purified water phantom using the SSEPI sequence. These results might be explained as follows. Purified water can result in stronger fluid convection in the phantom due to its lower viscosity compared with the mineral oil phantom. In addition, the SSEPI sequence might be more sensitive to the effect of fluid convection because of the very rapid acquisition compared with the FSE sequence in which the effect can be averaged out over a longer scan time. Consequently, the residual signals due to fluid convection in the phantom might overestimate the noise estimate in the subtraction method. Namely, the subtraction method may not be applicable to the SNR measurement in fluid phantoms with low viscosity using the SSEPI sequence. In contrast, the COMBET method is immune to the effects of fluid convection given that the signals from the purified water phantom are completely suppressed when both the diffusion gradient with the largest *b*‐value and the longest TE are used. In addition, the DELSET method resulted in substantially lower SNRs, especially using the SSEPI sequence because the T_2_ value of the phantom was longer than the longest TE, which caused the residual signals in the noise image. These results demonstrate the major advantage of the COMBET method over the DELSET method for long T_2_ tissues or materials. On the other hand, the much lower SNR using the FSE sequence compared to that obtained with the SSEPI sequence can be attributed to the fourfold higher spatial resolution (1 × 1 × 5 mm^3^ for the FSE vs. 2 × 2 × 5 mm^3^ for the SSEPI). Moreover, in the purified water phantom, the DELSET method appeared to be more closely comparable to the other methods using the FSE sequence compared to the SSPEI sequence, this finding might be explained by the above‐mentioned smaller residual signals in the noise image obtained using the DELSET because of the lower SNR of the FSE sequence. When the COMBET method is used, the signal intensity for the real part of the noise image of the purified water phantom was theoretically 3.11 × 10^−17^% for the SSEPI sequence and 3.41 × 10^−17^% for the FSE sequence under the imaging conditions used in the present study. These values are sufficiently below the signed 16‐bit quantization limit (1/32,768, i.e., 3.1 × 10^−3^% of the signal intensity at TE = 0 ms without a diffusion gradient) and thus support the validity of the noise image obtained using the COMBET method. Furthermore, the COMBET method resulted in no obvious differences in the SNRs between the diffusion gradient directions, indicating that the SNR is independent of the diffusion gradient axis. However, it should be noted that since these results pertain to the particular MRI system used in the present study and further validation of our findings in different MRI systems is warranted. The CVs of the SNR were generally comparable between the COMBET, DELSET, and subtraction methods. These results indicate that the repeatability of the SNR in the COMBET method is comparable with those in the other methods. Moreover, the SNR when the COMBET method was used had a stronger positive correlation with the effective slice thickness than the DELSET and subtraction methods, which indicates that the COMBET method may offer more robust SNR measurement than the other methods.

It was possible to obtain adequate noise and SNR maps in the head and upper abdomen using the COMBET method. The subtraction method is difficult to apply for SNR measurement of abdominal MRI because it requires two identical scans; thus, it is susceptible to image misregistration caused by motion. Moreover, the DELSET method is not applicable to SNR measurement of long T_2_ tissues, including the CSF and digestive fluid, because of the limited maximum TE available for the MRI systems. Compared with these methods, the COMBET method is more practical because it allows for simple and reliable SNR estimation even in long T_2_ tissues and does not require a specialized pulse sequence. As demonstrated in the present study, the COMBET method successfully addresses the main limitation of the DELSET method.

There were some limitations to this study. First, the COMBET method is only applicable to pulse sequences to which a diffusion gradient can be applied. Therefore, a feasible clinical application is the measurement of SNRs in diffusion imaging. Second, the largest available *b*‐value differs depending on the MRI systems. Although a larger *b*‐value can sufficiently attenuate signals in the noise image to reach the noise level, the optimal combination of *b*‐values and TEs for the COMBET method needs to be validated for different MRI systems and pulse sequences in future studies.

## 5 CONCLUSION

The COMBET method, which is suitable for pulse sequences to which a diffusion gradient can be applied, offers a simple and practical approach to SNR measurement that is applicable to long T_2_ tissues.

## AUTHOR CONTRIBUTIONS

Naoki Ohno: Conceptualization, methodology, software, validation, formal analysis, investigation, data curation, visualization, and writing‐original draft. Tosiaki Miyati: Conceptualization, methodology, and writing–review and editing. Hirotaka Oyabu: Conceptualization, methodology, formal analysis, investigation, data curation, visualization, and writing–original draft. Toshifumi Gabata: Supervision. Satoshi Kobayashi: Supervision.

## CONFLICT OF INTEREST

The authors declare no conflict of interest.
